# Dissociated repetition deficits in aphasia can reflect flexible interactions between left dorsal and ventral streams and gender-dimorphic architecture of the right dorsal stream

**DOI:** 10.3389/fnhum.2013.00873

**Published:** 2013-12-19

**Authors:** Marcelo L. Berthier, Seán Froudist Walsh, Guadalupe Dávila, Alejandro Nabrozidis, Rocío Juárez y Ruiz de Mier, Antonio Gutiérrez, Irene De-Torres, Rafael Ruiz-Cruces, Francisco Alfaro, Natalia García-Casares

**Affiliations:** ^1^Unit of Cognitive Neurology an Aphasia, Department of Medicine, Centro de Investigaciones Médico-Sanitarias, University of MalagaMalaga, Spain; ^2^Department of Psychosis Studies, Institute of Psychiatry, King's Health Partners, King's College LondonLondon, UK; ^3^Department of Psychobiology and Methodology of Comportamental Sciences, Faculty of Psychology, University of MalagaMalaga, Spain; ^4^Unit of Molecular Imaging, Centro de Investigaciones Médico-Sanitarias, General Foundation of the University of MalagaMalaga, Spain; ^5^Department of Medicine, Faculty of Medicine, University of MalagaMalaga, Spain

**Keywords:** transcortical motor aphasia, conduction aphasia, repetition, dual dorsal-ventral pathways, diffusion tensor tractography, positron emission tomography, functional magnetic resonance imaging

## Abstract

Assessment of brain-damaged subjects presenting with dissociated repetition deficits after selective injury to either the left dorsal or ventral auditory pathways can provide further insight on their respective roles in verbal repetition. We evaluated repetition performance and its neural correlates using multimodal imaging (anatomical MRI, DTI, fMRI, and^18^FDG-PET) in a female patient with transcortical motor aphasia (TCMA) and in a male patient with conduction aphasia (CA) who had small contiguous but non-overlapping left perisylvian infarctions. Repetition in the TCMA patient was fully preserved except for a mild impairment in nonwords and digits, whereas the CA patient had impaired repetition of nonwords, digits and word triplet lists. Sentence repetition was impaired, but he repeated novel sentences significantly better than clichés. The TCMA patient had tissue damage and reduced metabolism in the left sensorimotor cortex and insula. DTI showed damage to the left temporo-frontal and parieto-frontal segments of the arcuate fasciculus (AF) and part of the left ventral stream together with well-developed right dorsal and ventral streams, as has been reported in more than one-third of females. The CA patient had tissue damage and reduced metabolic activity in the left temporoparietal cortex with additional metabolic decrements in the left frontal lobe. DTI showed damage to the left temporo-parietal and temporo-frontal segments of the AF, but the ventral stream was spared. The direct segment of the AF in the right hemisphere was also absent with only vestigial remains of the other dorsal subcomponents present, as is often found in males. fMRI during word and nonword repetition revealed bilateral perisylvian activation in the TCMA patient suggesting recruitment of spared segments of the left dorsal stream and right dorsal stream with propagation of signals to temporal lobe structures suggesting a compensatory reallocation of resources via the ventral streams. The CA patient showed a greater activation of these cortical areas than the TCMA patient, but these changes did not result in normal performance. Repetition of word triplet lists activated bilateral perisylvian cortices in both patients, but activation in the CA patient with very poor performance was restricted to small frontal and posterior temporal foci bilaterally. These findings suggest that dissociated repetition deficits in our cases are probably reliant on flexible interactions between left dorsal stream (spared segments, short tracts remains) and left ventral stream and on gender-dimorphic architecture of the right dorsal stream.

## Introduction

In their pioneering studies on aphasia Broca (1861, 1863) and Wernicke (1874, 1906, 1977) described distinct syndromes associated with involvement of anterior and posterior cortical areas of the left hemisphere, respectively. One of these syndromes was chiefly characterized by reduced speech fluency with relatively spared auditory comprehension (Broca's aphasia) (Lazar and Mohr, 2011), whereas in the other syndrome spontaneous speech was abundant but abnormal in content and auditory comprehension was also impaired (Wernicke's aphasia) (Albert et al., 1981). At the same time, Lichtheim (1885) was investigating language deficits which apparently resulted from selective damage to major commissures linking cortical speech areas. Lichtheim (1885) was particularly interested in two contrasting syndromes which in his view resulted from selective damage to commissural pathways. One such syndrome, early described by Wernicke is what nowadays is known as conduction aphasia (CA) (Benson et al., 1973; Henderson, 1992), whereas the other, termed by Lichtheim “inner commissural aphasia,” is currently known as transcortical motor aphasia (TCMA) (a term coined by Wernicke) or dynamic aphasia (Luria and Tsvetkova, 1968; Albert et al., 1981; Berthier, 1999; Robinson et al., 2008). From a clinical viewpoint, TCMA and CA are easily distinguished between them by the ability to repeat language. Abnormal repetition, fluent spontaneous speech and preserved auditory comprehension are the key features of CA (Benson et al., 1973; Albert et al., 1981; Goodglass, 1992). By contrast, a relative preservation of repetition in the face of nonfluent verbal output and preserved auditory comprehension defines TCMA (Albert et al., 1981; Freedman et al., 1984; Berthier, 1999). These two syndromes result from the involvement of different structures. The typical TCMA syndrome has been linked with left frontal lesions beneath the Broca's area disconnecting it from the supplementary motor area (Albert et al., 1981; Freedman et al., 1984; Cauquil-Michon et al., 2011). Typical TCMA may also occur in association with extensive damage to the left superior frontal gyrus involving the supplementary motor area (Alexander and Schmitt, 1980). Variants of TCMA (Freedman et al., 1984; Taubner et al., 1999) and DA^1^ (see Robinson et al., 2008) usually involve the left anterior perisylvian language cortex (Brodmann's areas 44, 45, 4, 6, anterior insula) although DA can also follow bilateral striatocapsular lesions (Gold et al., 1997; patient BS in Berthier, 1999, pp. 69–74). Left temporoparietal or inferior parietal lesions with variable involvement of the AF and insular cortex induce CA (Benson et al., 1973; Damasio and Damasio, 1980).

**Figure 1:**
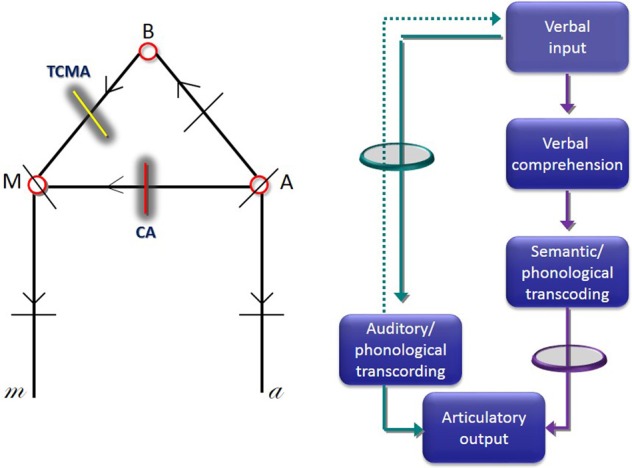
**Lichtheim's first diagram of speech centers, commissural pathways and predicted sites of lesions that would cause aphasia (left image)**. In this diagram, “A” indicates the center of auditory images, “M” the center of motor images, and “B” the center of concepts. Lesions interrupting the commissure “A”-“M” cause conduction aphasia (CA) (red line), whereas interruption of the commissure interconnecting B-M causes transcortical motor aphasia (TCMA) (yellow line). Diagram depicting a two route model to repetition (right image) (adapted from McCarthy and Warrington, 1984). The green circle represents the route that should be interrupted to induce CA, whereas the purple circle represents a lesion in the connection that should be interrupted to induce TCMA.

Wernicke, Lichtheim and their contemporary scholars described the surface features and pathological correlates of both syndromes. Wernicke noted that interruption of white matter pathways would lead to abnormal repetition, but in his original formulation of CA repetition deficits were not included as a hallmark component of the syndrome (Geschwind, 1967; Henderson, 1992; De Bleser et al., 1993). Wernicke (1874) provided the first two-route model of language repetition and this diagram was further elaborated by Lichtheim (1885) (Figure 1). He envisioned that repetition could be impaired by disruption of the route linking the auditory images of words (“A”) and motor images of words (“M”) which passes through the insular cortex. Despite having abnormal repetition, Lichtheim further contended that patients presenting with this disorder would maintain volitional speech relatively preserved because it could be mediated by direct connections between the concept center (“B”) and the center of motor images (“M”). This syndrome corresponds to classical CA (Geschwind, 1965). In complimentary terms, Lichtheim (1885) interpreted his “inner commissural aphasia” (TCMA) as resulting from the interruption of the connection between concept center (“B”) and the center for motor images of words (“M”) (Berthier, 1999). Although further elaborations on the routes engaged in speech repetition were advanced (Kussmaul, 1877; Heilman et al., 1976), the cognitive mechanisms underpinning speech repetition in these syndromes remained unexplored until the seminal study by McCarthy and Warrington (1984) (hereafter McC & W). They performed a comprehensive evaluation of speech production deficits in two patients with CA (ORF and RAN) and in another one (ART) with TCMA which revealed a double dissociation in tasks that manipulated semantic processing requirements of repetition. McC & W did find that in tasks requiring active semantic processing, repetition performance was facilitated in the two patients with CA and hindered in the TMCA patient. Indeed, ORF and RAN performed better repeating semantically meaningful word triplet lists than similar lists with no semantic relatedness and they also repeated novel sentences (e.g., “She went to buy some milk”) better than clichéd sentences (e.g., “On top of the world”). The opposite pattern of dissociation was found in ART in whom repetition performance was facilitated by tasks which entail little or no semantic processing (repetition of clichés) and rely more on automatic online strategies. Results from this investigation, allowed McC &W to reappraise the two-route model for speech production early championed by Lichtheim (1885) (Figure 1) (see further details below). Nevertheless, McC & W concluded “.., the biological necessity and *modus operandi* of dual processing routes in speech production remain obscure” (p. 482). Regrettably, as a result of the limited development of brain imaging by that time, McC & W were unable to dissect the neural architecture of white matter pathways underpinning repetition.

With the advent of diffusion tensor imaging (DTI) an accurate *in vivo* delineation of white matter tracts is being achieved (Mesulam, 2005; Catani and Mesulam, 2008; Saur et al., 2008; Turken and Dronkers, 2011; Weiller et al., 2011; Rijntjes et al., 2012; Cloutman, 2013) and this fruitful knowledge has prompted retrospective speculations on the respective role of different components of the arcuate fasciculus (AF) in these two contrasting syndromes, CA and TCMA (Catani et al., 2005). Catani et al. (2005) attempted to provide an anatomical explanation for McC & W's findings by focusing their analysis on the involvement of the direct and indirect segments of the AF in CA and TCMA. These researchers noted that lesions in the temporoparietal cortex in both CA patients (ORF and RAN) extended deeply to injure the direct long segment of the AF (the classical AF) responsible for fast, automatic repetition of words and nonwords, but spared the indirect segment engaged in active semantic processing required for novel sentence repetition. This may account not only for impaired repetition and sparing of spontaneous speech and auditory comprehension, but also for better performance on repetition of novel sentences over over-learned clichés. By contrast, Catani et al. (2005) also noted that the inferior parietal lesion in ART was more superficial than the ones in ORF and RAN and hence ideally suited to injure only the superior part of the indirect segment or its parietal cortical relay station fully preserving the direct long segment. This strategically placed lesion in ART may have hindered repetition of novel sentences by preventing the access to meaning during repetition but his ability to repeat over-learned phrases (clichés) requiring less cognitive processing and effort was preserved because the direct segment of the AF remained intact. Lesion locations in patients reported by McC & W were established using computerized tomography scans thus hampering establishing reliable anatomo-functional relationships. Therefore, although the *a posteriori* interpretations of McC & W's findings by Catani et al. (2005) represent a step forward into the mechanisms underlying repetition, further studies using modern imaging methods are warranted.

Testing brain-damaged patients with different repetition deficits and selective injury to either the left dorsal or ventral white matter bundles can provide valuable insight not only on their respective roles in verbal repetition, but also on the vicarious capacity of right white matter tracts to compensate repetition deficits. However, the selective involvement of a single white matter pathway (Carota et al., 2007) or discrete portions of the cerebral cortex (Vallar et al., 1997) after highly focal lesions is exceptional because brain lesions are usually large and rarely respect anatomical boundaries. In the present study, we took advantage of the exceptional circumstance of examining two aphasic patients with dissociated speech production and repetition deficits and small contiguous but non-overlapping perisylvian infarctions. We evaluated the anatomo-functional correlates of these dissociated deficits with multimodal imaging.

## Materials and methods

### Participants

#### Patient RTP

RTP was a 41-year-old female who had completed 8 years of formal education and worked as a secretary before the stroke. She was referred to our unit for evaluation and treatment of a chronic nonfluent post-stroke aphasia. Her past history was unremarkable until December 2007 when she suffered the spontaneous rupture of a saccular aneurism of the left anterior choroid artery. The aneurism was successfully blocked with coiling, but 1 week after the procedure RTP suddenly developed mutism with preserved comprehension and right hemiparesis with sensory loss mostly affecting the hand. By that time, she also had dysphagia and oral apraxia. An angiography disclosed vasospasm of the left middle cerebral artery (M2 and M3 segments) and a brain MRI showed a small infarct involving the left sensorimotor cortex with extension into the middle and posterior insular cortex. After hospital discharge, she received speech-language therapy for 1 year. On formal evaluation performed in April 2009, RTP had a TCMA and a discrete right hand sensorimotor deficit. Although her communication in activities of daily living was judged to be relatively spared by their relatives, she did not attempt to communicate spontaneously and only speak when addressed. Spontaneous speech was sparse and hesitant. She had long latency to initiate verbal emissions, but her messages were devoid of paraphasias, perseverations, agrammatism or articulatory deficits. Auditory comprehension was impaired only for understanding complex sequential commands. Repetition and naming were preserved and were considerably better than spontaneous speech. Writing was impaired. She also had moderate cognitive and motor slowness, depression, mild apathy and reduced quality of life, particularly in physical and communication domains.

#### Patient JGG

JGG was a 52-year-old male who had completed 12 years of formal education and ran his own business before the stroke. He was referred to our unit for evaluation and treatment of a chronic fluent post-stroke aphasia. He had a history of treated hypothyroidism and well-controlled hypertension. On January 2009 while on vacations in Bangkok (Thailand) he suddenly lost consciousness and felt. On awakening, he was admitted to a local hospital where naming and short-term memory problems were identified. An MRI angiogram revealed a complete occlusion of the M4 segment of the left middle cerebral artery and an anatomical MRI showed a small left temporoparietal infarct. A cavum septum pellucidum and a cavum vergae were also seen (DeLisi et al., 1993; Choi et al., 2008). On returning to Spain, he received 3 months of speech-language therapy and some improvement in aphasia severity were noted. On a formal evaluation performed in August 2009, JGG showed language deficits consistent with a mild reproduction CA (Shallice and Warrington, 1977; Nadeau, 2001). His spontaneous speech was fluent and well-articulated but he made unsuccessful self-corrective attempts in barely accessible words during spontaneous speech (*conduite d'ecart*). Comprehension and object naming were virtually intact. Repetition was preserved for short words but not for polysyllabic words and sentences. He was depressed and mildly apathetic showing reduced communication in activities of daily living. He also felt tired and developed right fronto-parietal headache soon after starting aphasia testing or therapy, a set of symptoms resembling the ones reported in patients with minor strokes in either left parietal, thalamic or caudate regions (Staub and Bogousslavsky, 2001; Radman et al., 2012; Tang et al., 2013).

### Handedness

Handedness was evaluated using the Edinburgh Inventory of Handedness (EIH) (Oldfield, 1971). Both patients were right handed (EIH, above +40). RTP was strongly right handed (EIH +100) and JGG also performed most activities, including writing, with the right hand but he used either hand to do some activities (EIH +77). Both patients had a negative history for familial left-handedness, prenatal or perinatal injuries, learning disabilities or developmental disorders.

### Language assessment: aphasia profile

#### Methods

Language deficits were assessed with the oral subtests (spontaneous speech, comprehension, repetition, and naming) of the Western Aphasia Battery (WAB) (Kertesz, 1982) to obtain an Aphasia Quotient (AQ), a measure of aphasia severity. On the WAB-AQ patients are considered to have aphasia when they score <93.8 and lower scores indicate more severe deficits. The Naming × Frequency subtest of the Psycholinguistic Assessments of Language Processing in Aphasia (PALPA 54) (Kay et al., 1992; Valle and Cuetos, 1995) was also administered.

#### Results

Both patients obtained WAB-AQs below the cut-off score for diagnosis of clinically significant aphasia (Kertesz, 1982). RTP obtained a moderately impaired WAB-AQ (74.9/100) with mild impairment on auditory comprehension (8.7/10), repetition (8.4/10) and naming (8.3/10) and severe reduction of verbal output (fluency in spontaneous speech score: 4/10), a combination of deficits that characterizes the TCMA syndrome (Kertesz, 1982; Berthier, 1999). However, the profile of TCMA in RTP was atypical because she had deficits in nonword repetition and a lesion in the central perisylvian area (Freedman et al., 1984; Berthier, 1999)^**1**^. According to recent accounts (Robinson et al., 1998, 2005, 2008) the profile of aphasia in RTP could be also classified as *mixed dynamic aphasia*^**1**^ because she had phonological impairments (impaired nonword repetition) besides the severely reduced spontaneous speech (see below). JGG obtained a better WAB-AQ (84/100) than RTP and his subtest scores were mildly impaired for fluency (8/10), comprehension (9.3/10) and naming (9.1/10). His score on repetition (8/10) though impaired in polysyllabic words and sentences was better than the repetition scores (range: 0–6.9) required in the WAB (Kertesz, 1982) for meeting the diagnosis of CA. Although the WAB classified language deficits in JGG as anomic aphasia, his object naming was intact. Moreover, the WAB taxonomic criteria are not sensitive enough to distinguish different mild aphasic syndromes. Therefore, the aphasic deficits in JGG were classified as a mild reproduction CA (Nadeau, 2001). Picture naming was normal in both patients obtaining normal scores on WAB-object naming subtest (RTP: 59/60; JGG: 60/60) and PALPA 54 (both 59/60).

### Speech production

#### Methods

Since measures to rate spontaneous speech (fluency and information content) of the WAB have limited reliability, other rules for rating verbal production during picture description in aphasia are commonly used (Nicholas and Brookshire, 1993; Berndt et al., 2000; Marchina et al., 2011; Zipse et al., 2012). Therefore, communicative informativeness and efficiency of connected speech were evaluated with speech samples obtained during the description of the “Picnic Scene” from the WAB (time limit: 5 min). All descriptions were audiotaped. Speech samples were transcribed and analyzed for percentage of correct information units (CIUs) defined as non-redundant content words that convey correct information about the stimulus (Nicholas and Brookshire, 1993) using the following formula: number of CIUs/number of words × 100. According to Nicholas and Brookshire (1993) to be classified as CIUs, words should be not only intelligible in context, but also accurate, relevant and informative with respect to the stimulus. Meaningless utterances, perseverations, paraphasias and other inappropriate information (exclamations) were counted as words, but not classified as CIUs.

#### Results

RTP had reduced informativeness and overall efficiency of speech measured with CIU and %CIU in comparison to JGG (Table 1). Spontaneous speech in RTP was hesitant and perseverative as reflected by the high number of pauses (>3 s.) which in part contributed to reduced fluency. Spontaneous speech in JGG was fluent and more informative than in RTP but his utterances were occasionally punctuated by phonemic approximations to the target word (*conduite d'approche*).

**Table 1:** **Language, communication, and behavior**.

**Test**	**RTP**	**JGG**
Fluency in spontaneous speech (WAB)	4	8
**PICTURE DESCRIPTION (WAB)**		
Correct information units	48	74
Percentage of correct information units	46	92.5
Pauses (>3 s)	16	1
Semantic fluency (animal naming—WAB)	6	11
Naming × Frequency (PALPA 54) (max: 60)	59	59
**COMMUNICATIVE ACTIVITY LOG**		
Quality of communication (max: 90)	83	69
Amount of communication (max: 90)	68	59
Total (max: 180)	151	128
Stroke aphasia depression questionnaire (max: 84)	31	43

WAB indicates Western Aphasia Battery; PALPA, Psycholinguistic. Assessments of Language Processing in Aphasia.

### Communication

#### Method

Communication in activities of daily living was assessed with the Communicative Activity Log (CAL) (Pulvermüller and Berthier, 2008). The CAL is composed of 36 questions divided in two parts that address quality of communication (e.g., “How well would the patient verbally express criticisms or make complaints?”) and amount of communication (e.g., “How frequently would the patient verbally express criticisms or make complaints?”). The CAL quality of communication score is obtained by summing up scores for items 1–18. The amount of communication score is obtained by summing up scores over items 19–36. The total score range from 0 to 180 and high scores indicate better everyday communication. The CAL was completed by a reliable family member in the presence of one member of the research team in order to clarify potential misunderstanding in questions' content or scoring.

#### Results

Assessment of communication of daily living revealed that both patients were impaired in both quality and amount of communication (Table 1). However, RTP obtained better scores than JGG. Her quality of communication was rated much better than the amount of communication, whereas JGG obtained similar low scores in both communication subscales.

### Behavioral evaluation: depression

#### Method

Depression was assessed with the Stroke Aphasic Depression Questionnaire (SADQ) (Sutcliffe and Lincoln, 1998). The SADQ is a 21-item questionnaire developed based on observable behaviors commonly associated with depressed mood. Questions address mood (e.g., “Does he/she have weeping spells?”), social interaction, loss of interest, sleep-related problems, and motivation. The SADQ was completed by a reliable family member on behalf of the patient behavior and high scores indicate more severe depression.

#### Results

Both patients had symptoms of post-stroke depression with JGG obtaining higher scores on the SADQ than RTP (Table 1).

### Experimental repetition testing

Although both patients obtained relatively similar scores on the WAB-repetition subtest, qualitative differences were found. Repetition in RTP was flawless except for the longest sentence which she reproduced incompletely most likely due to a mild short-term memory deficit. Repetition in JGG was impaired for polysyllabic words and sentences. Therefore, further tests were administered. Patients' scores on repetition of words, nonwords and word triplets were compared with those from a group of 14 healthy controls [5 men and 9 women; mean age: 57.1 ± 6.6 years (range: 47–67 years); education: 10.2 ± 3.7 years; range: (6.5–18 years)] (Berthier, 2001).

### Word and nonword repetition

#### Methods

The two patients were asked to repeat a list of auditorily presented words (*n* = 80) taken from the Frequency Dictionary of Spanish Words (Juilland and Chang-Rodriguez, 1964) and nonwords (*n* = 80). The corpus of words (nouns) included 20 high-frequency/high-imageability nouns, 20 high-frequency/low-imageability nouns, 20 low-frequency/high-imageability nouns and 20 low-frequency/low-imageability nouns. Nonwords contained 3–8 letters, were pronounceable, and most of them (71%) were derived from real words. All responses were audiotaped for later transcriptions and only exact repetitions were scored as correct.

#### Results

Repetition was normal for words (nouns) and impaired for nonwords [RTP: χ^2^_(1)_, 15.53, *p* < 0.0001; JGG: χ^2^_(1)_, 51.57, *p* < 0.0001]. RTP repeated nonwords significantly better than JGG [χ^2^_(1)_, 22.01, *p* < 0.0001] (Table 2).

**Table 2:** **Repetition of words, nonwords, and digits**.

**Test**	**RTP**	**JJG**	Healthy control subjects^*^
Words (*n* = 80)	80 (1.0)	77 (0.95)	79.8 ± 0.54 (range 79–80)
Nonwords (*n* = 80)	64 (0.80)	34 (0.42)	78.7 ± 1.2 (range 76–80)
**GRAMMATICAL CLASS**			
Nouns (*n* = 60)	60 (1.0)	58 (0.97)	59.9 ± 0.26 (range: 39–40)
Verbs (*n* = 50)	49 (0.98)	48 (0.96)	50 ± 0
Adjectives (*n* = 50)	48 (0.96)	47 (0.94)	50 ± 0
Functors (*n* = 40)	40 (1.0)	40 (1.0)	39.9 ± 0.26 (range: 39–40)
Digits (forward)	4	3	Not tested
**REPETITION OF WORD TRIPLETS AND SENTENCES**			
**High frequency**			
Random	19/20 (0.95)	11/20 (0.55)	19.0 ± 0.8 (range: 17–20)
Loosely constrained	19/20 (0.95)	8/20 (0.40)	18.7 ± 1.0 (range: 17–20)
Constrained	19/20 (0.95)	15/20 (0.75)	19.4 ± 0.6 (range: 18–20)
Total	57/60 (0.95)	33/60 (0.55)	–
**Low frequency**			
Random	17/20 (0.85)	1/20 (0.05)	17.0 ± 2.5 (range: 11–20)
Loosely constrained	17/20 (0.85)	5/20 (0.25)	18.6 ± 1.3 (range: 16–20)
Constrained	20/20 (1.0)	12/20 (0.60)	18.7 ± 1.2 (range: 16–20)
Total	54/60 (0.90)	18/60 (0.30)	
Clichés^*^	40/40 (1.0)	25/40 (0.62)	Not tested
Novel phrases^*^	37/40 (0.92)	35/40 (0.87)	Not tested

* Experimental tests from Berthier (2001). See further details in text.

### Repetition: grammatical class

#### Method

In order to identify possible differences in the ability to repeat grammatical categories of words, the patients were asked to repeat a list of 200 words composed of nouns (*n* = 60), verbs (*n* = 50), adjectives (*n* = 50), and functors (*n* = 40) which were selected from the Frequency Dictionary of Spanish Words (Juilland and Chang-Rodriguez, 1964).

#### Results

Both patients showed a near perfect performance on this test (Table 2).

### Digit production

#### Method

This was assessed with the Digit Production/Matching Span (PALPA 13) which assesses the immediate serial recall of sequence of digits (2–7) of increased length. In the Matching Span patients are required to indicate among two alternatives the sequence verbally presented by the examiner if the sequence is identical or not. For this task, a sequence of digits (2–7) of increased length was presented.

#### Results

Both patients had restricted digit production (Table 2). Digit span matching was not evaluated.

### Repetition of word triplets

#### Method

To assess the influence of lexical-semantic information on repetition ability when the demand of the auditory-verbal short-term memory is increased both patients were asked to repeat word triplet lists. This task is a modification of the one used by McCarthy and Warrington (1984, 1987) in patients with CA. In our battery two sets of 60 three-word lists (verb-adjective-noun) were created (Berthier, 2001). These were composed of word strings of increasing semantic richness that is from non-organized to organized semantic information. Two 20 three-word lists (List 1: 60 high-frequency words; List 4: 60 low-frequency words) consisted of random word combinations (e.g., “buy-sweet-country”). Two other 20 three-words lists (List 2: 60 high-frequency words; List 5: 60 low-frequency words) conveyed loosely constrained meaningful information (e.g., “shake-full-bottle”), and two other 20 three-word lists (List 3: 60 high-frequency words; List 6: 60 low-frequency words) conveyed closely constrained meaningful information (e.g., “cut-lovely-flower”). Words were read at a rate of one per second and patients were required to repeat the words in the order given by the examiner. Responses were scored for the number of lists repeated verbatim in each condition.

#### Results

Performance on this task was normal in RTP and markedly impaired in JGG patients (Table 2). RTP repeated significantly better high-frequency word triplets than JGG [χ^2^_(1)_, 3.86, *p* = 0.049], whereas there were no significant differences in the repetition of low-frequency triplets [χ^2^_(1)_, 2.86, *p* = 0.091]. RTP repeated different word triplets with similar efficiency and JGG showed better performance on repetition of high-frequency and low-frequency with constrained semantic information than other word triplets but differences did not reach statistical significance. However, when high-frequency and low-frequency word triplets were analyzed together, JGG repeated word triplets containing constrained semantic information significantly better (27/40,0.67) than word triplets with random semantic organization [12/40,0.30; χ^2^_(1)_, 9.68, *p* < 0.005] and loosely semantic organization [13/40,0.32; χ^2^_(1)_, 8.34, *p* < 0.005].

### Repetition of clichés and novel sentences

#### Method

To explore possible dissociation between both types of sentences, both patients were asked to repeat well-known Spanish clichés (*n* = 40) taken from the 150 Famous Clichés of Spanish Language (Junceda, 1981) as well as a set of novel sentences (*n* = 40) that were construed following the methodology described by Cum and Ellis (1999) and Berthier et al. (2011).

#### Results

RTP repeated significantly better clichés than JGG [χ^2^_(1)_, 15.88, *p* < 0.0001] but there were no differences between them in the ability to repeat novel sentences. JGG repeated novel sentences significantly better than clichés [χ^2^_(1)_, 5.33, *p* = 0.021], whereas RTP repeated clichés and novel sentences with similar efficiency (Table 2).

## Multimodal neuroimaging

### Structural MRI

#### Methods

MRIs studies in both patients were performed on a 3-T magnet (Philips Gyroscan Intera, Best, The Netherlands) equipped with an eight-channel Philips SENSE head coil. Head movements were minimized using head pads and a forehead strap. High-resolution T-1 structural images of the whole brain were acquired with three dimensional (3D) magnetization prepared rapid acquisition gradient echo (3 D MPRAGE) sequence (acquisition matrix: 240/256 r; field of view: 240 ms; repetition time [TR]: 9.9 ms; echo time [TE]: 4.6 ms; flip angle: 8; turbo field echo (TFE) factor: 200; 1 × 1 × 1 mm^3^ resolution). One hundred eighty two contiguous slices, each 1-mm thick, 0 mm slice gap, were acquired. The total acquisition time of the sequence was about 4:24 min. In addition to the 3D MPRAGE, a standard axial T-2 weighted/FLAIR (*TR* = 11.000 ms; *TE* = 125/27 ms; 264 × 512 matrix; field of view [*FOV*] = 230 × 230; 3-mm-thick slices with 1 mm slice gap) was obtained. A Short TI Inversion Recovery (STIR) was used to produce 24, 2.5 mm axial slices (interslice gap = 1 mm; *TR* = 4718 ms; *TE* = 80 ms; inversion time = 200 ms; 264 × 512 matrix; *FOV* = 230 mm; number of excitations = 2). Lesion volumes were manually drawn by one of us (SFW) who was blind to patients' aphasic profiles. The drawings were made using MRIcro software (Rorden, 2005; http://www.mccauslandcenter.sc.edu/mricro/mricro/) on T_1_-weighted images.

#### Results

**Figure 2:**
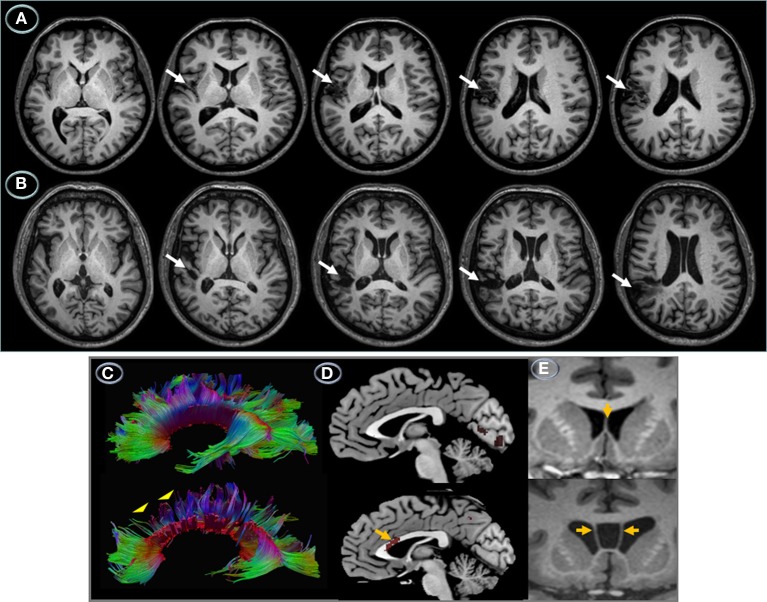
**Left hemisphere structural lesions**. Axial T_1_-weighted MRI showing a small infarction in the patient with transcortical motor aphasia **(A)** involving the left sensorimotor cortex and medial insula (white arrows). The lesion is superficial sparing the deep white matter. The MRI in the patient with conduction aphasia shows a small infarction involving the left posterior temporal gyrus and supramarginal gyrus extending deeply into the lateral ventricle wall **(B)** (white arrows). The left hemisphere is represented on the left side of the images. DTI, MRI, and PET of the corpus callosum. Brain imaging of the corpus callosum. Midsaggital DTI **(C)** and ^18^FDG-PET images **(D)** and coronal T_1_-weighted MRI **(E)** of the corpus callosum in patients RTP (top panel) and JGG (bottom panel). In RPT the corpus callosum was structurally **(C)** and functionally intact **(D)**. Although RTP had an infarction in the left sensorimotor cortex, this did not result in noticeable changes in the anterior segments of the corpus callosum. The rostral body of corpus callosum in JGG shows diminished streamlines **(C)**, bottom panel (yellow arrowheads) and decrement of metabolic activity **(D)**, bottom panel (yellow arrow). The fact that structural and functional involvement of the anterior corpus callosum in JGG does not interrupt fibers interconnecting the damaged left temporoparietal cortex with its homologous in the right hemisphere suggests that corpus callosum involvement was unrelated to the stroke lesion. Anatomical MRIs show normal septum pellucidum (grade 0, normal) (yellow arrow) in RTP **(E)**, top panel and an enlarged cavum septum pellucidum (grade 4, severe) (Degreef et al., 1992; DeLisi et al., 1993; Kim and Peterson, 2003) in JGG (yellow arrows) **(E**, bottom panel**)**.

Anatomical MRI revealed contiguous but non-overlapping perisylvian infarctions (Figure 2). Lesions were small and had similar volumes (*RTP* = 16.5 cm^3^; *JGG* = 15.8 cm^3^). Tissue damage in RTP involved the left sensorimotor cortex (precentral gyrus and postcentral gyrus). There were minute foci in the left medial/posterior insula and superior temporal gyrus, but most part of this gyrus and the whole supramarginal gyrus were spared. JGG had tissue damage centered in the left posterior temporal gyrus and supramarginal gyrus. The cortical lesion in RTP was more superficial than the one found in JGG which extended deeply to reach the ventricular wall at the level of the white matter underlying the superior temporal gyrus and supramarginal gyrus. There were no lesions in the right hemisphere. The MRI in JGG additionally disclosed cavum septum pellucidum and cavum vergae, but no other developmental malformations (Figure 2).

### Diffusion tensor imaging (DTI)

#### Methods

DTI allows for “*in vivo*” measurement of the diffusive properties of water in a way that allows information to be garnered about the microstructural organization of tissue (Basser et al., 1994). Tractography enables the orientation of white matter (WM) to be ascertained, thus making possible the segregation of WM into separate sections based on the paths of the distinct tracts (Le Bihan, 2003). Data acquisition was performed using multi-slice single-shot spin-echo echo-planar imaging (EPI) with specific parameters as follows: FOV 224 mm, 2-mm-thick slices with 0 mm slice gap, *TE* = 117 ms, *TR* = 12408 ms, and b factor: 3000 s/mm2. The EPI echo train length consisted of 59 actual echoes reconstructed in a 112 × 128 image matrix. Sixty four diffusion directions were used in order to allow for precise construction of the diffusion tensor. Motion and eddy current correction were performed using FSL's FDT (http://www.fmrib.ox.ac.uk/fsl/) eddy current correction tool (Smith et al., 2004; Woolrich et al., 2009). Diffusion tensor estimation was carried out in using Diffusion Toolkit's least-square estimation algorithm for each voxel (Ruopeng Wang, Van J. Wedeen, TrackVis.org, Martinos Center for Biomedical Imaging, Massachusetts General Hospital). The whole brain tractography used an angular threshold of 35° and an FA threshold of 0.2. The tensor was spectrally decomposed in order to obtain its eigenvalues and eigenvectors. The fiber direction is assumed to correspond to the principal eigenvector (the eigenvector with the largest eigenvalue). This vector was color coded (green for anterior-posterior, blue for superior-inferior and red for left-right) in order to help generate the color FA map. An FA map was also generated from these eigenvalues. This too was done using Diffusion Toolkit. Virtual dissections of the three parts of the AF, the corpus callosum and the inferior frontal-occipital fasciculus/extreme capsule (IFOF/EmC) were performed by using a region of interest (ROI) approach, following the directions of a white matter tractography atlas (Catani and Thiebaut de Schotten, 2012) and Catani et al. (2007). All virtual dissections were performed using TrackVis (Ruopeng Wang, and Van J. Wedeen, TrackVis.org, Martinos Center for Biomedical Imaging, Massachusetts General Hospital).

#### Results

**Figure 3:**
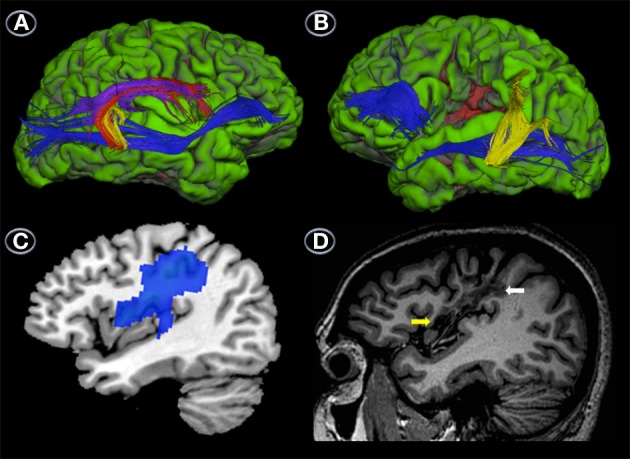
**Uninflated surface of the right (A) and left hemispheres (B) (FreeSurfer reconstruction) of the patient with transcortical motor aphasia (RTP) showing gyri cultured in green with sulci shown in red**. DTI of right hemisphere perisylvian pathways superimposed on RTP anatomical MRI shows well-developed temporo-frontal, temporo-parietal and parieto-frontal streams (red, yellow, and purple, respectively) and ventral stream (blue) **(A)**, whereas in the left hemisphere only the parieto-temporal segment of the arcuate fasciculus (yellow) and part of the ventral stream (blue) could be reconstructed **(B)**. This image also shows the location of the infarct in the lower pericentral region (red area). Parasaggital T_1_-weighted MRI view of the left hemisphere shows the infarction in the sensorimotor cortex (white arrows) and medial insula (yellow arrow) **(C)**. Parasaggital view of the ^18^FDG-PET (MRIcroN) showing an area of reduced metabolic activity in the left fronto-insular region (blue) **(D)** which is slightly larger than the area of infarction depicted in **(C)**. See further details in Table 4.

Reconstruction of the three segments of the AF and the IFOF/EmC revealed differences between the patients in both the primarily affected (left) and unaffected (right) hemispheres. Reconstruction of the AF of the right hemisphere revealed that RTP had well-developed temporo-frontal, temporo-parietal and parieto-frontal sections (Figure 3A). The IFOF/EmC was also intact (Figure 3A). In the left hemisphere, however, we were only able to reconstruct the temporo-parietal segment of the AF (Figure 3B), with the parieto-frontal and temporo-frontal segments seemingly destroyed by the lesion (Figure 3B). We were also unable to reconstruct the entire path of the left IFOF/EmC in RTP (Figure 3B). The occipito-temporal and frontal sections were reconstructed separately, but the section of this tract that swings from the temporal lobe through to the external capsule and connects both parts shown in Figure 3B was affected by the lesion and unreconstructable.

**Figure 4:**
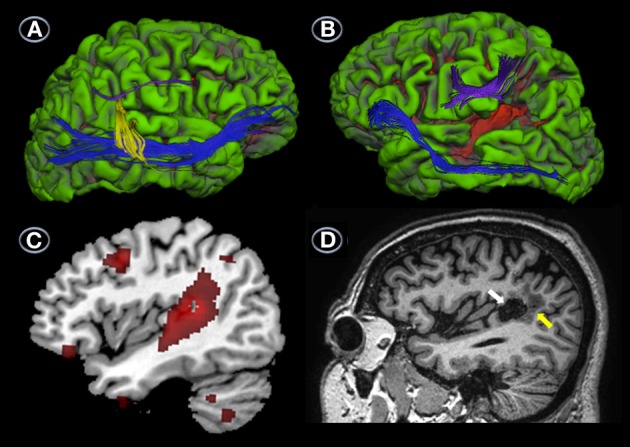
**DTI, MRI, and PET**. Uninflated surface of the right **(A)** and left hemispheres **(B)** (FreeSurfer reconstruction) of the patient with conduction aphasia (JGG) showing gyri cultured in green with sulci shown in red. DTI of right hemisphere perisylvian pathways superimposed on JJG anatomical MRI shows a complete lack of the direct temporo-frontal section of the AF in the right hemisphere. The indirect temporo-parietal (yellow) and parieto-frontal (purple) segments are present but very small. White matter tract reconstruction in the left hemisphere shows that the fronto-parietal segment of the AF (purple) was intact, but the temporo-parietal and temporo-frontal segments were unreconstructable, and seemingly destroyed by the lesion **(B)**. The ventral stream (inferior frontal-occipital fasciculus/extreme capsule) was reconstructed successfully in both hemispheres **(A,B)**. The surface image of the left hemisphere depicted in **(B)** also shows the infarction (red area) involving the posterior-superior and middle temporal gyri and part of the supramarginal gyrus. Parasaggital T_1_-weighted image shows a component of the infarction in the supramarginal gyrus (white arrow) surrounded by perinecrotic tissue (yellow arrow) **(C)**. Parasaggital image of the ^18^FDG-PET (MRIcroN) showing an area of reduced metabolic activity in the left temporoparietal region (red) **(D)** which is slightly larger at the level of the posterior temporal gyrus than the area of infarction depicted in **(C)**. Less voluminous foci of reduced metabolic activity (all in red) are also shown in the left middle frontal gyrus (Brodmann's area 6), lateral orbitofrontal cortex (Brodmann's area 11), inferior temporal gyrus (Brodmann's area 20), and cerebellum **(D)**. See further details in Table 4.

Analysis of the same tracts in JGG revealed a complete lack of the direct temporo-frontal section of the AF in the right hemisphere, as is often found in males (Catani et al., 2007; Figure 4A). The indirect temporo-parietal and parieto-frontal sections were present but very small (Figure 4A). Tractography of the left hemisphere tracts revealed that the fronto-parietal segment of the AF was intact, but the temporo-parietal and temporo-frontal segments were unreconstructable, and seemingly destroyed by the lesion (Figure 4B). The IFOF/EmC was reconstructed successfully in both hemispheres (Figures 4A,B).

Reconstruction of the corpus callosum of each patient revealed a large and completely intact tract in RTG (Figure 2C top panel), while a large section of the midbody of the corpus callosum in JGG revealed only a sparse amount of reconstructed streamlines with the rostral midbody particularly affected (Figure 2C, bottom panel) (complimentary data is shown in positron emission tomography section).

### Functional MRI

#### Methods

The fMRI included the following parameters: T_2_-weighted fMRI scans were acquired using a gradient echo FFE-EPI (fast-field echo-echo planar image) sequence (repetition time/echo time = 2500/30 ms, flip angle = 60, field of view = 23.0 × 23.0 cm, matrix = 96 × 128; 40 axial slices aligned parallel to the anterior commissure-posterior commissure line, slice thickness = 2.5 mm; interslice gap = 0.5 mm). fMRI data processing and pre-processing were carried out using FEAT (FMRI Expert Analysis Tool) Version 5.98, part of FSL (Version 4.1.8) (Jenkinson et al., 2012). Functional datasets underwent pre-processing using the specifications outlined below. Data was corrected for movement during the scan using mcFLIRT (Jenkinson et al., 2002). BET brain extraction was used to delete non-brain tissue from functional datasets as preparation for registration to structural images (Smith, 2002). Data were “prewhitened” and smoothed to a standard of 5 mm FWHM. High-pass filter cut-off 60 s (as per FSL recommendation of a filter equal to the total design cycle time). Low resolution functional images were first registered to each individual's brain-extracted high resolution structural image using a linear search (6 DOF). Highres structurals were then registered to the standard space MNI-152 T1 2 mm template with a 12 DOF linear transformation followed by a non-linear warp. Manual denoising of data was completed by examining FSL MELODIC (Beckmann and Smith, 2004) output generated for each subject during pre-processing. Components were deleted if they demonstrated activations correlated with subject movement or other artifacts rather than authentic activation to task according to recommendations set out in (Kelly et al., 2010). Time-series statistical analysis was carried out using FILM with local autocorrelation correction (Woolrich et al., 2001). Z (Gaussianised T/F) statistic images were thresholded using clusters determined by Z > 5 and a (corrected) cluster significance threshold of *p* = 0.05.

### Stimuli and experimental design

Both patients performed the behavioral testing (repetition of words, nonwords and word triplets) and fMRI on the same day. All paradigms contained the same number of stimuli in the behavioral session and fMRI session and stimuli were presented in the same order and timing. The experimental paradigms included three covert repetition activation tasks. In the single item repetition paradigm, one task contained 40 high-frequency, concrete Spanish nouns [e.g., “casa” (house)] whereas the other task contained 40 nonwords which were derived from real words [“piedra” (stone) → *pierla*] by substituting the phonemes on the basis of Spanish phonotactical rules. The third paradigm was a word triplet repetition task which contained 20 three-high frequency words composed of semantically random word combinations (e.g., “buy-sweet-country”). Only high-frequency words were used and were taken from the Frequency Dictionary of Spanish Words (Juilland and Chang-Rodriguez, 1964). All tasks were binaurally presented through headphones and patients were requested to covertly repeat the item or triplet without delay. During the word and nonword paradigms 4 baselines and 4 stimulus sequences were presented and each period (baselines and stimulus sequences) lasted 30 s. Each stimulus sequence included 10 items with a presentation time of 3.0 s per stimulus. The same methodology was applied for word triplet repetition, but each stimulus sequence included 5 word triplets with a presentation rate of 6.0 per stimulus. There was no repetition of words, nonwords or triplets between periods or time points. The number and proportion of errors (including errors and no responses) in each tasks during the behavioral testing was analyzed in each patient.

### Behavioral and imaging results

**Figure 5:**
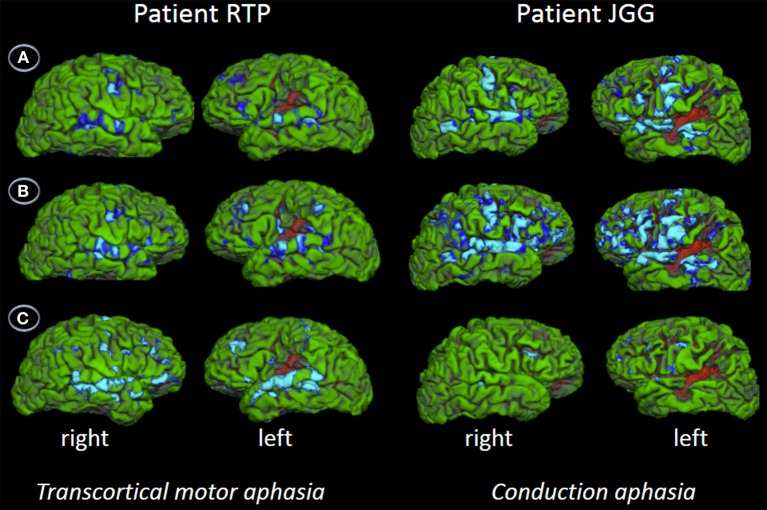
**Functional MRI**. The figures show the contrast words **(A)**, nonwords **(B)**, and word triplets **(C)** versus rest. Contrasts are shown on patients' uninflated cortical surface of the right and left hemispheres and significant activations (*p* < 0.05, corrected) are depicted in light blue and blue. The fMRI shows bilateral perisylvian activation in both patients in all three tasks. Although JGG obtained lower performance in word and nonword repetition tasks than RTP, he showed greater areas of activation, extending into motor, premotor and prefrontal areas in addition to the perisylvian areas activated by both of them. In contrast, word triplet repetition activated a greater bilateral network in RTP than JGG. See further details in text and Table 3.

Behavioral assessment of single word repetition was flawless (40/40, 1.0) in RTP and almost perfect (38/40, 0.95) in JGG (χ^2^_(1)_, 0.51, *p* = 0.447) but nonword repetition was abnormal in both patients with RTP performing significantly better (33/40, 0.80) than JGG (17/40, 0.42) [χ^2^_(1)_, 11.85, *p* < 0.001]. Word triplet repetition was normal in RTP (38/40, 0.95) and moderately impaired in JGG (22/40, 0.55) (χ^2^_(1)_, 14.81, *p* < 0.000). The fMRI showed bilateral perisylvian activation in both patients in all three tasks. These are shown in Figure 5 and Table 3. JGG showed greater areas of activation, extending into motor, premotor and prefrontal areas in addition to the perisylvian areas activated by both patients during the word and nonword repetition tasks (Figure 5). In contrast, word triplet repetition activated a greater bilateral network in RTP than JGG, with JGG exhibiting focal activation in left frontal and superior temporal areas, and small right-sided superior temporal sulcus and inferior frontal gyrus (Figure 5).

**Table 3:** **Brain areas activated on functional Magnetic Resonance Imaging during repetition tasks**.

**Patients**	**Region**	**Side**	**MNI coordinates**			**Cluster size**	
			***x***	***y***	***z***	***z***	**Vox**
**WORD REPETITION**							
RTP	Inferior frontal gyrus/insula/temporal pole	L	−48	14	−4	8.98	3105
	Anterior cingulate/supplementary motor area		−2	6	40	8.79	1997
	Cerebellum	R	20	−74	−48	8.39	1739
	Superior temporal gyrus	R	72	−22	0	8.6	1267
	Precentral gyrus	R	58	2	34	8.94	746
	Inferior frontal gyrus	R	42	16	24	8.64	691
JGG	Precentral gyrus/inferior frontal gyrus	R	54	2	46	12.1	7042
	Superior temporal gyrus	L	−56	−26	4	12.4	6239
	Occipital lobe		−6	−72	10	10.6	2377
	Supplementary motor area/anterior cingulate		−10	4	74	11.3	1954
	Cerebellum	L	−34	−60	−28	10.7	1105
**NONWORD REPETITION**							
RTP	Superior temporal gyrus	L	−64	−14	4	8.9	6471
	Middle/superior temporal gyrus	R	52	−34	−2	8.99	3809
	Anterior cingulate/supplementary motor area		0	6	44	8.67	2055
	Angular gyrus	R	38	−52	44	8.32	560
JGG	Inferior frontal gyrus/superior temporal gyrus/middle temporal gyrus/precentral gyrus/postcentral gyrus	L	−56	24	18	11.5	6447
	Middle temporal gyrus/superior temporal gyrus/inferior frontal gyrus/middle frontal gyrus/precentral gyrus	R	66	−30	0	11.6	5543
	Supplementary motor area/anterior cingulate		0	4	64	11.1	1887
	Occipital lobe		20	−64	4	9.96	1352
**WORD TRIPLET REPETITION**							
RTP	Superior temporal gyrus	L	−54	−36	2	9.39	1505
	Middle/superior temporal gyrus	R	64	−20	−4	9.13	814
	Frontal pole	R	30	52	−16	9.58	388
	Temporal pole/inferior frontal gyrus	R	56	12	−6	8.06	122
JGG	Middle temporal gyrus	L	−52	−48	2	7.33	338
	Superior temporal gyrus	L	−56	−28	2	7.01	201
	Precentral gyrus	L	−48	6	36	6.91	163
	Inferior frontal gyrus	L	−56	14	−2	7.28	156
	Angular gyrus	R	62	−46	44	6.02	143
	Inferior frontal gyrus	L	−56	24	18	6.78	140
	Inferior frontal gyrus	R	54	26	20	6.44	132
	Frontal pole	L	−32	50	16	5.83	102

Localization is based on stereotactic coordinates MNI (Montreal Neurological Institute). These coordinates refer to the location of maximal activation indicated by the Z score in a particular anatomic structure. Distances are relative, to the intercommissural (AC-PC) line in the horizontal (x), anteriorposterior (y) and vertical (z) directions.

### Positron emission tomography

#### Methods

A [18F]-fluorodeoxyglucose positron emission tomography (^18^FDG-PET) was performed at rest using a GE Advance PET/CT scanner (GE Medical Systems). Preparation for the study included fasting for at least 6 h before the administration of ^18^F-FDG and oral hydration with water. Both patients and 25 healthy control subjects (female/male: 11/14; mean age ± *SD*; 56.9 ± 5.7 years; age range: 45–67 years) refrained from drinks containing alcohol, caffeine, and smoking for 12 h before the PET scan. The subjects received an approximate dose of [18F] FDG 370 MBq at resting conditions with eyes closed and in an environment with dimmed ambient light. Forty minutes after the injection PET acquisition were performed in a GE Discovery ST PET scanner during 20 min in 3D mode with a field of view of 15.7 cm and a pixel size 2.3 mm, after CT for attenuation correction purposes. The images were reconstructed with iterative construction resulting in 47 sections with a slice thickness of 3.27 mm. Statistical parametric mapping software (SPM5, http://www.fil.ion.ucl.ac.uk/spm/software/spm5/), based on MATLAB, v7.7 (The Mathworks Inc, Natick, MA), was used for realignment, transformation into standard stereotactic space, smoothing (6 mmFWHM), and statistical analyses. Individual global counts were normalized by proportional scaling to a mean value of 50 mg/100 ml/min.

#### Results

**Figure 6:**
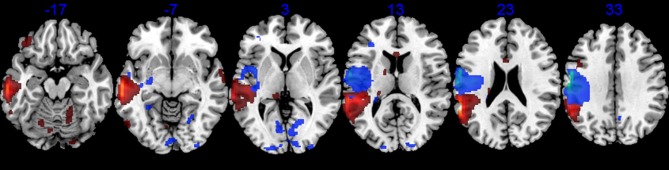
**^18^FDG-PET**. Axial PET images (MRIcroN) of patients with transcortical motor aphasia (blue) and conduction aphasia (red) showing significant reductions of metabolic activity in the left perisylvian areas and interconnected areas (see Table 4). Note that although the structural lesion in RTP was more superficial than the one in JGG (see Figure 2), decreased metabolic activity extended deeply to affect white matter in both cases. The left hemisphere is represented on the left side of the images.

The areas of reduced metabolic activity in both patients are shown in Table 4 and Figures 2, 3, 4, 6. The PET in RTP showed areas of significantly decreased metabolic activity in the area of infarction and surrounding it in the left precentral gyrus, postcentral gyrus, insula, and middle frontal gyrus. Other cortical areas in both cerebral hemispheres together with the left caudate also showed decreased metabolic activity. In JGG significant decrements of metabolic activity were found in the area of structural damage and surrounding it in the left supramarginal gyrus, middle temporal gyrus, inferior temporal gyrus and in the middle frontal gyrus. Decreased glucose metabolism was also found in other cortical areas in both cerebral hemispheres, left posterior thalamus, left caudate, bilateral cerebellum and rostral body of the corpus callosum (Figures 2, 6).

**Table 4:** **Brain regions showing significant decreases of metabolic activity in RTP and JGG relative to 25 healthy control subjects**.

**RTP**							**JGG**						
**Region**	**Side**	**MNI coordinates**			**Cluster size**		**Region**	**Side**	**MNI coordinates**			**Cluster size**	
		***x***	***y***	***z***	***z***	**Vox**			***x***	***y***	***z***	***z***	**Vox**
Precentral gyrus(BA6)	L	−52	−12	31		59.39	Supramarginal gyrus (BA40)	L	−60	−44	27		45.22
Postcentral gyrus(BA2)	L	−44	−29	40		26.22	Middle temporal gyrus (BA21)	L	−65	−27	−9		42.99
Insula(BA13)	L	−38	−14	21	7.82	17.32	Supramarginal gyrus (BA40)	L	−58	−36	41		35.85
Paracentral lobule(BA6)	R	11	−29	44	6.67	11.57	Middle frontal gyrus (BA9)	L	−44	16	35	7.56	15.71
Paracentral lobule(BA5)	R	9	−42	50	5.17	7.12	Middle frontal gyrus (BA11)	L	−38	38	−25	7.36	14.64
Middle occipital gyrus(BA19)	R	15	−90	9	6.44	10.74	Inferior semi-lunar lobule	R	44	−71	−42	7.15	13.63
Lingual gyrus(BA18)	L	−5	−84	−6	6.31	10.28	Pyramis	R	18	−79	−28	6.90	12.50
Middle occipital gyrus(BA18)	L	−14	−91	6	6.10	9.62	Cerebellar tonsil	R	51	−61	−34	6.86	12.34
Middle frontal gyrus(BA10)	L	−29	27	14	5.93	9.08	Middle frontal gyrus (BA6)	L	−33	17	51	6.81	12.16
Parahippocampal gyrus(BA19)	L	−29	−43	−1	5.80	8.72	Cerebellar tonsil	R	−42	−69	−41	6.25	10.10
Lingual gyrus(BA18)	L	−7	−69	4	5.67	8.36	Pyramis	R	−18	−90	−30	5.96	9.19
Lingual gyrus(BA19)	R	17	−62	1	5.64	8.29	Pyramis	R	−18	−81	−28	5.58	8.12
Posterior cingulate(BA30)	R	4	−65	8	5.59	8.14	Inferior temporal gyrus (BA20)	L	−51	−4	−41	6.24	10.05
Paracentral lobule(BA5)	L	−11	−35	44	5.58	8.12	Inferior occipital gyrus (BA17)	R	19	−93	−4	6.15	9.77
Caudate (tail)	L	−24	−31	17	5.48	7.86	Parahippocampal gyrus (BA35)	R	20	−27	−12	5.97	9.21
Caudate (tail)	L	−27	−36	12	5.42	7.72	Middle occipital gyrus (BA19)	L	−56	−67	−1	5.94	9.13
Precuneus(BA7)	R	20	−56	43	5.45	7.79	Thalamus (pulvinar)	L	−14	−31	1	5.86	8.90
Middle frontal gyrus(BA10)	L	−32	42	14	5.34	7.52	Thalamus (pulvinar)	L	−22	−25	14	5.73	8.54
Precuneus(BA7)	R	4	−57	31	5.30	7.42	Precuneus (BA7)	R	6	−53	51	5.75	8.57
							Declive	R	−7	−78	−9	5.72	8.50
							Lingual gyrus (BA18)	L	−5	−76	−3	5.28	7.38
							Cerebellar tonsil	R	22	−48	−45	5.71	8.46
							Middle temporal gyrus (BA21)	R	71	−11	−9	5.68	8.38
							Superior temporal gyrus (BA22)	R	65	1	−11	5.66	8.35
							Anterior cingulate (BA24)	L	1	24	9	5.64	8.29
							Declive	R	−20	−69	−13	5.64	8.29
							Declive	R	−27	−82	−14	5.31	7.44
							Inferior temporal gyrus (BA20)	L	−60	−56	−10	5.57	8.11
							Caudate (body)	L	−16	2	12	5.48	7.86
							Declive	R	18	−67	−14	5.30	7.42
							Declive	R	23	−56	−15	5.27	7.35

FEW p < 0.05 (corrected for multiple comparisons). Localization is based on stereotactic coordinates MNI (Montreal Neurological Institute). These coordinates refer to the location of maximal decrement metabolic activity indicated by the Z score in a particular anatomic structure. Distances are relative, to the intercommissural (AC-PC) line in the horizontal (x), anterior-posterior (y) and vertical (z) directions.

## Discussion

In this study, we examined the structural and functional correlates of speech production and repetition in two patients with contrasting aphasic syndromes, TCMA and CA. In their cognitive investigation of the two routes of speech production, McC & W (1984) reported a double dissociation in patients with CA and TCMA (see Introduction). They did find that repetition relying on active semantic processing (novel sentences) was facilitated in CA and hindered in TMCA, whereas repetition that minimized the engagement of semantics (clichés) was privileged in TCMA and hampered in CA. We replicated and extended these behavioral findings and subsequent elaborations by other authors (Catani et al., 2005; Catani and Thiebaut de Schotten, 2012) on the plausible neural correlates of this double dissociation in the aphasics described by McC & W (1984). From a clinical and radiological standpoint, our cases are not fully comparable with the ones described by McC & W (1984) because their patients had more severe aphasias and larger lesions than those found in our cases. Moreover, one of the two patients with CA (ORF) and the patient with TCMA (ART) by McC & W (1984) were examined in the acute post-stroke period (~1 or 2 months post-onset). By contrast, our patients were evaluated in the chronic stage, thus implying the possibility of recovery of certain functions. Nonetheless, we trust that our patients are comparable with McC & W's cases (1984) in other respects, since all these patients had a clear-cut double dissociation in tasks that manipulated semantic processing requirements of repetition. This piece of evidence may inform how selective damage or sparing of different segments of the dorsal stream (AF) and of the ventral stream (IFOF/EmC) can underpin stable deficits or preserved/compensated function.

Large-scale cortico-subcortical networks are engaged in language function (Mesulam, 1998), but the respective role of cortical areas and white matter tracts as well as their dynamic interaction in subserving language repetition is still controversial (Saur et al., 2008; Bernal and Ardila, 2009; Rolheiser et al., 2011; Turken and Dronkers, 2011; Berthier et al., 2012; Dick and Tremblay, 2012; Rijntjes et al., 2012; Friederici and Gierhan, 2013). Verbal repetition is a multifaceted function which involves multiple domains (attention, phonological working memory, and lexical-semantic, syntactic, phonemic and motor production processes) requiring the concerted action of several cortical areas and white matter bundles in both cerebral hemispheres (Price et al., 1996; Castro-Caldas et al., 1998; Burton et al., 2001; Collete et al., 2001; Abo et al., 2004; Saur et al., 2008; Majerus et al., 2012; Hartwigsen et al., 2013). While performance in language repetition tasks in healthy subjects is almost perfect, differences between subjects have been described (Castro-Caldas et al., 1998; Catani et al., 2007) which may depend on demographic factors (e.g., gender, literacy) and on individual variability in the anatomy and function of cortical areas and white matter tracts (Catani et al., 2005; Berthier et al., 2012). The neural basis of repetition is currently interpreted in a dual dorsal-ventral pathways framework (Hickok and Poeppel, 2007; Rauschecker and Scott, 2009; Axer et al., 2013; Cloutman, 2013; Friederici and Gierhan, 2013; Kümmerer et al., 2013). The ventral pathway connects the frontal and temporal cortices via the ventral stream (IFOF/EmC and uncinate fasciculus) and it has been associated with comprehension processes by mapping sounds onto meaning (Makris and Pandya, 2009; Turken and Dronkers, 2011; Rijntjes et al., 2012; Axer et al., 2013). The AF is formed by three segments including the long-direct pathway connecting the temporal cortex to the prefrontal cortex, the indirect segment composed of the anterior segment connecting the inferior parietal cortex and Broca's area and a posterior segment connecting temporal and parietal regions (Catani et al., 2005). The direct segment supports sensory-to-motor mapping participating in speech perception and fast, automatic repetition (Catani et al., 2005; Hickok and Poeppel, 2007; Saur et al., 2008; Friederici, 2012), whereas the indirect segment has been linked to verbal comprehension (semantic/phonological transcoding, complex syntactic processing) (Catani et al., 2005; Friederici and Gierhan, 2013).

While the ventral streams are symmetrical, the dorsal streams (AF) are more asymmetrical (Paus et al., 1999; Catani et al., 2005; Turken and Dronkers, 2011; Axer et al., 2013) and most DTI studies reveal a gender-dimorphic architecture of the dorsal stream (Catani et al., 2005; Catani and Mesulam, 2008; Häberling et al., 2013, but see Gharabaghi et al., 2009). Leftward biased asymmetry of the AF predominates in males and usually coexists with the absence or vestigial development of its long segment in the right hemisphere. By contrast, females tend to have more symmetrical patterns (Catani et al., 2005; Powell et al., 2006; Catani and Mesulam, 2008; Thiebaut de Schotten et al., 2011; Catani and Thiebaut de Schotten, 2012; Häberling et al., 2013). The leftward biased asymmetry of the AF may depend on genetic influences (Häberling et al., 2013) and also on repeated practice (Halwani et al., 2011). The ventral streams are present at birth (Perani et al., 2011) but the segment of the AF connecting the temporal cortex and Broca's area is undetectable in newborns (Perani et al., 2011) and matures late adopting the adult-like structure by the age of 7 years (Friederici, 2012). Thereafter, the macrostructure (tract length and width) and microstructure (fractional anisotropy) of the AF do not remain static; rather the AF is gradually modeled by skill acquisition and repeated practice (e.g., musicians, bilinguals) throughout the lifespan (Draganski and May, 2008; Halwani et al., 2011; May, 2011; Mackey et al., 2012; Schlegel et al., 2012) and also by intensive training tailored to remediate pathological conditions (aphasia) (Schlaug et al., 2009, 2010; Breier et al., 2011; Zipse et al., 2012). Based on these behavioral and imaging data, we suggest that dissociated performances in speech production (nonfluent in TCMA and fluent in CA) and repetition tasks (preserved in TCMA and impaired in CA) in the present cases resulted from damage to the left dorsal stream and gender-dimorphic architecture of their right dorsal streams.

### Dissociated speech production deficits

Speech production deficits (fluency) in our cases were dissociated: slowness and hesitation pinpointed spontaneous speech in the TCMA patient (RPT), whereas fluent utterances occasionally punctuated by self-corrections were heard in the CA patient (JGG). Picture description was more informative in JGG than in RTP though both produced meaningful utterances devoid of paraphasias, articulatory or apraxic deficits. Auditory comprehension and object/picture naming were largely preserved in both cases, but word list generation (animal naming) was poor with RPT producing less exemplars than JGG. The fact that both patients had small lesions of similar volumes makes unlikely that this variable could explain the differences found in speech production (Lazar et al., 2008; Marchina et al., 2011). Instead, we attribute dissociated speech production deficits to differences in both lesion location and metabolic changes in regions nearby and distant to the areas of infarction. Anatomical MRIs in our patients showed small contiguous but non-overlapping left perisylvian infarctions. In RTP, there was involvement of left sensorimotor cortex and medial insula relevant for planning and execution of speech (Riecker et al., 2005; Sörös et al., 2006; Ackermann and Riecker, 2010; Price, 2010), whereas fluent speech in JGG could be related to involvement of the left temporoparietal cortex with sparing of more anterior cortical areas. Pervasive and sometimes long-lasting deficits in speech production can also emerge in association with damage to left hemisphere white matter tracts (medial subcallosal fasciculus, periventricular substance) (Naeser et al., 1989) and lesion load in the left AF can also impair speech production in aphasia (Marchina et al., 2011). Of note, DTI revealed damage to different segments of the AF in our cases which might account for dissociated speech production deficits. RTP had damage to the left parietofrontal segment of the AF already related to impaired speech fluency in TCMA (Catani et al., 2005; Catani and Thiebaut de Schotten, 2012), whereas this segment could be identified in patient JGG with fluent CA.

An important caveat on the contribution of the AF lesion load to speech production deficits in our cases is the concomitant cortical involvement. On the basis of recent research, it could be argued that functional and structural damage to the sensorimotor cortex, pars opercularis, and insula in RTP impacted fluency (Blank et al., 2002; Borovsky et al., 2007) more than the temporoparietal involvement in JGG (Hickok et al., 2011). On the other hand, the cortical temporoparietal component of the lesion in JGG could actually be responsible of other deficits characteristic of CA since it was strategically placed to disrupt phonological short-term memory (Vallar et al., 1997; Leff et al., 2009; Buchsbaum et al., 2011) and auditory-motor integration dependent upon the activity of the area Spt, a small region recently implicated in pathogenesis of CA (for review see Hickok et al., 2011). Indeed, some researchers argue that the left temporoparietal involvement suffice to explain language and short-term memory deficits in CA (Buchsbaum et al., 2011; Hickok et al., 2011), thus undermining the traditional “disconnection” mechanism in JGG (Geschwind, 1965). However, PET data in JGG showed significant metabolic decrements in frontal areas (Brodmann's areas 6, 9) remote to the temporoparietal region but connected with it via the AF (Rilling et al., 2008). Moreover, it should be kept in mind that the cortical damage encompassing the area Spt and surrounding regions also involve the cortical origins of the AF and this coupled with the additional involvement of the AF at subcortical level may have impacted on the activity of distant areas. In other words, damage of one component of a network may alter the function of the whole system (Gratton et al., 2012; Rijntjes et al., 2012).

### Communication and behavior

We also did find reductions in everyday verbal communication with the TCMA patient (RTP) unexpectedly attaining better scores in the quality and amount of communication subscales of the CAL (Pulvermüller and Berthier, 2008) than the CA patient (JGG). These findings appear paradoxical particularly in light of recent brain imaging studies reporting that deficits in speech fluency and conversational speech co-occur because the responsible lesions overlap in cortical (Borovsky et al., 2007) and subcortical sites (Marchina et al., 2011). The notion that communication relies on language is intuitively appealing, but recent studies suggest that language and communication may be dissociable abilities by virtue of being reliant on the activity of different neural systems (Ellis et al., 2005; Willems and Varley, 2010; Coelho et al., 2012; Moreno-Torres et al., 2013). The processing of phonology, syntax and lexical-semantics depends on components (perisylvian cortex, temporal pole) of an extended neural network that dynamically interacts with other networks (e.g., medial prefrontal cortex, basal ganglia, cerebellum) engaged in complimentary functions including the motivation to communicate messages, understanding the intention of others and so forth (Willems and Varley, 2010). Therefore, if some components of these large-scale networks are lesioned whereas other components remain functional, evaluation of language and communication abilities in such cases will show dissociable deficits (Ellis et al., 2005; Willems and Varley, 2010; Moreno-Torres et al., 2013).

In our cases, RTP exhibited the predictable correspondence between diminished speech production and poor communication. Verbal communication deficits in RTP affected more the amount than the quality of communication perhaps because the prefrontal areas (Brodmann's areas 9, 10, and 46) and the anterior insula implicated in narrative discourse (Alexander, 2006; Moreno-Torres et al., 2013) were only mildly affected. By contrast, the amount and quality of communication were equally affected in JGG and neuroimaging (anatomical MRI, DTI, PET) disclosed involvement of structures implicated in communication including the inferior parietal lobe (Geranmayeh et al., 2012), left anterior cingulate gyrus, rostral body of corpus callosum, bilateral cerebellum and right paravermis (Durisko and Fiez, 2010; Marvel and Desmond, 2010; Willems and Varley, 2010). The fact that both patients were depressed probably contributed to reduced functional communication in socially interactive contexts (Fucetola et al., 2006).

### Dissociated repetition deficits

Multimodal brain imaging findings in our cases extend the interpretation of traditional models (Lichtheim, 1885; Wernicke, 1906; McCarthy and Warrington, 1984) and ensuing elaborations (Catani et al., 2005) by incorporating the compensatory activity of other white matter tracts and cortical areas. Our results suggest that dissociated repetition deficits in our cases depend on available interactions between left dorsal stream (spared segments, short tracts remains) and left ventral stream as well as on gender-dimorphic architecture of the right dorsal stream. In the TCMA patient (RTP), damage to the left sensorimotor cortex and insula extending into the dorsal stream and part of the ventral stream did not alter repetition, except for a moderate impairment in nonword and digit repetition. The abnormal performance of RTP on nonword repetition after damage of the dorsal stream is compatible with its putative role on phonological transcoding (Saur et al., 2008; Rijntjes et al., 2012; Cloutman, 2013), but this function was only moderately impaired in RTP thus implying additional mediation or compensation by other structures. Moreover, preserved performance on word and sentence repetition tasks as documented in RTP is highly unlikely after damage to the left dorsal stream unless other structures also contribute to these language functions. DTI of the left hemisphere showed that the temporo-parietal segment of the left AF was spared as well as its intertwining with the ventral stream in the posterior temporal lobe (Rijntjes et al., 2012). Complimentary fMRI data during all repetition tasks showed a consistent activation of the left middle and superior temporal regions where the temporo-parietal segment of the left dorsal stream and the ventral stream interact (Rolheiser et al., 2011; Rijntjes et al., 2012; Cloutman, 2013). Word, word lists (triplets) and sentence repetition was almost intact in RTP with no influence of linguistic variables (word frequency, imageability, lexicality, meaningfulness of word triplets, and familiarity of sentences) thus raising the possibility that in the face of an unavailable left parieto-frontal and temporo-frontal AF segments, this verbal information was redirected via its spared temporo-parietal segment to the ventral stream to be repeated successfully (see Lopez-Barroso et al., 2011). Moreover, we also attribute the successful repetition performance of RTP to the contribution of the right dorsal and ventral streams (Berthier et al., 2012). In this regard it should be noted that RTP had well-developed right dorsal and ventral streams and that during fMRI tasks there was a bilateral activation in frontal areas suggesting recruitment of left and right dorsal streams and transmission of signals to superior temporal cortices indicating a shift of activation from dorsal stream to ventral stream. RTP was a female with apparently symmetric dorsal streams, an anatomical pattern which correlates with better verbal learning through word repetition in females than in males with leftward biased asymmetry of the dorsal stream (Catani et al., 2007). Accordingly, it is possible that the topography of fMRI activation foci in RTP indicates a rather symmetric organization of repetition before the stroke (Berthier, 1999), or its reorganization in the right hemisphere after brain injury. The later possibility seems unlikely because the left hemisphere lesion in RTP was small and compensation by the right hemisphere after stroke usually takes place in cases with large left hemisphere lesions (Heiss and Thiel, 2006; Berthier et al., 2011; Turkeltaub et al., 2011).

Brain-behavior relationships in the CA patient (JGG) were different to that found in RTP. He was moderately impaired in repeating nonwords, digits, non-meaningful word triplets and clichés although he could repeat fairly well-words, meaningful word triplets and novel sentences. Multimodal brain imaging disclosed tissue damage and reduced metabolic activity in the left posterior temporal cortex/supramarginal gyrus with additional metabolic decrements in the left frontal lobe and other structures (see below). DTI showed the left temporo-parietal and temporo-frontal segments of the AF interrupted by the lesion, but the both ventral streams were spared. Importantly, the direct segment of the AF in the right hemisphere was also absent with only vestigial remains of the other dorsal subcomponents present, an architecture prevailing in males (Catani et al., 2005; Catani and Mesulam, 2008). Although both patients had small lesions of similar volumes, the fMRI tasks showed larger areas of bilateral perisylvian activation in JGG than in RTP extending into motor, premotor and prefrontal areas in addition to the perisylvian areas activated during the word and nonword repetition tasks by both of them. In contrast, word triplet repetition activated a greater bilateral network in RTP than JGG, with JGG exhibiting focal activation in left frontal and superior temporal areas, and small right-sided superior temporal sulcus and inferior frontal gyrus. This limited activation was not totally unexpected as word triplets were semantically unrelated and he could only repeat semantically-related three word strings. In the same context, JGG repeated novel sentences requiring active semantic processing significantly better that overlearned clichés, a dissociated performance suggestive of reliance on the ventral streams.

The activation of large areas in JGG was not observed in previous CA cases with small (Fernandez et al., 2004; case JVA in Berthier et al., 2012) and large structural lesions (Harnish et al., 2008). Rather, this activation resemble the patterns recently described in both normal children with still undeveloped AF (Brauer et al., 2010) and adolescents with early damage to AF (Yeatman and Feldman, 2013). The MRI in JGG disclosed enlarged cavum septum pellucidum/cavum vergae and involvement of the anterior corpus callosum besides the poorly developed right dorsal stream. A small cavum septum pellucidum (Grades 0–2) is considered a normal neuroanatomical variation and can occur in around 30% of healthy control subjects (DeLisi et al., 1993; Hopkins and Lewis, 2000; Choi et al., 2008). By contrast, an enlarged cavum septum pellucidum (Grades 3 and 4) represents a midline malformation and a marker of arrested development of neighboring structures such as hippocampus, septal nuclei, limbic system, or corpus callosum (Kim and Peterson, 2003; Brown et al., 2009). The presence of an enlarged cavum septum pellucidum has been associated with various disorders related to dysfunction of the aforementioned structures such as schizophrenia (Degreef et al., 1992; Trzesniak et al., 2012), bipolar disorder (Kim et al., 2007), obsessive-compulsive disorder (Chon et al., 2010), and developmental disorders (macro/microcephaly, mental retardation, developmental delay, Tourette syndrome) (Schaefer et al., 1994; Kim and Peterson, 2003). JGG had a negative history for these disorders and the presence of enlarged cavum septum pellucidum/cavum vergae was clinically unsuspected, yet their occurrence raises the possibility that other brain regions were abnormally developed as well. Embryologic development of the septum pellucidum is intimately associated with the corpus callosum and we found that this commissural pathway was fully normal in RTP but abnormal in JGG. DTI and PET in JGG showed sparse amount of reconstructed streamlines and reduced metabolic activity in the rostral body of the corpus callosum, respectively. Since this part of the corpus callosum interconnects premotor, motor and supplementary motor regions (Witelson, 1989; Aboitiz and Montiel, 2003; Hofer and Frahm, 2006; Saur et al., 2010), it is possible that reduced inter-hemispheric interactions coupled with undeveloped right dorsal stream explained the limited capacity of JGG to compensate repetition deficits. The results obtained in JGG should be interpreted with caution because he had minor developmental anomalies that probably interfered with the development and maturation of some brain regions. Although these malformations were clinically silent and might be interpreted as incidental MRI findings, their impact in the profile and evolution of aphasic deficits remains to be determined.

The study of the neural correlates of dissociated speech production and repetition deficits with multimodal imaging in these cases confronted us with a complex scenario characterized by reorganization of repetition in both cerebral hemispheres. Our findings are preliminary because they were documented only in two patients and because performing single-subject experimental research using neuroimaging (DTI, fMRI, PET) entails some disadvantages in comparison with case series studies and group studies (Kiran et al., 2013). Although further studies are clearly needed, our findings in two well-matched patients with contrasting aphasic syndromes suggest that dissociated repetition deficits in these aphasic syndromes are probably reliant on flexible interactions between spared components of the left dorsal and ventral streams and on gender-dimorphic architecture of the right dorsal stream.

### Conflict of interest statement

Marcelo L. Berthier declares association with the following companies: Bayer, Eisai, Eli Lilly, GlaxoSmithhKline, Janssen, Merz, Novartis, Nutricia, Pfizer, and Lundbeck. Rocío Juárez y Ruiz de Mier declares association with Pfizer. Seán Froudist Walsh, Guadalupe Dávila, Alejandro Nabrozidis, Antonio Gutiérrez, Irene De-Torres, Rafael Ruiz-Cruces, Francisco Alfaro, and Natalia García-Casares declare that the research was conducted in the absence of any commercial or financial relationships that could be construed as a potential conflict of interest.

## Footnotes

^1^Transcortical motor aphasia (TCMA) is a heterogeneous condition and cases with atypical or “near variant” features have been described. These cases are atypical because some speech deficits (impaired articulation, stuttering) and language deficits (impaired auditory comprehension) deviate from the classical pattern, denoting the extension of the typical left frontal subcortical lesion to its neighboring areas in the frontal operculum, motor area, basal ganglia/internal capsule, or insula (Freedman et al., 1984; Taubner et al., 1999). Dynamic aphasia (DA) is closely allied with TCMA and is also heterogeneous in its main features and lesion sites (Berthier, 1999). Two main DA types have been described, pure DA (reduced verbal output without other language impairments) and mixed DA (reduced verbal output with additional phonological, lexical, syntactical and/or articulatory impairments) associated with mild and moderate involvement of the anterior perisylvian area, respectively, (Robinson et al., 1998, 2005, 2008).
